# Longitudinal multi-modal muscle-based biomarker assessment in motor neuron disease

**DOI:** 10.1007/s00415-019-09580-x

**Published:** 2019-10-17

**Authors:** Thomas M. Jenkins, James J. P. Alix, Jacob Fingret, Taniya Esmail, Nigel Hoggard, Kathleen Baster, Christopher J. McDermott, Iain D. Wilkinson, Pamela J. Shaw

**Affiliations:** 1 Sheffield Institute for Translational Neuroscience, University of Sheffield, 385a Glossop Road, Sheffield, S10 2HQ UK; 2 Department of Neurology, Sheffield Teaching Hospitals NHS Foundation Trust, Sheffield, UK; 3 Departments of Neurophysiology, Sheffield Teaching Hospitals NHS Foundation Trust, Sheffield, UK; 4 Academic Unit of Radiology, University of Sheffield, Sheffield, UK; 5 Statistics Services Unit, School of Mathematics and Statistics, University of Sheffield, Sheffield, UK

**Keywords:** Muscle, MRI, Motor neuron disease, Amyotrophic lateral sclerosis, MUNIX

## Abstract

**Background:**

Clinical phenotypic heterogeneity represents a major barrier to trials in motor neuron disease (MND) and objective surrogate outcome measures are required, especially for slowly progressive patients. We assessed responsiveness of clinical, electrophysiological and radiological muscle-based assessments to detect MND-related progression.

**Materials and methods:**

A prospective, longitudinal cohort study of 29 MND patients and 22 healthy controls was performed. Clinical measures, electrophysiological motor unit number index/size (MUNIX/MUSIX) and relative T2- and diffusion-weighted whole-body muscle magnetic resonance (MR) were assessed three times over 12 months. Multi-variable regression models assessed between-group differences, clinico-electrophysiological associations, and longitudinal changes. Standardized response means (SRMs) assessed sensitivity to change over 12 months.

**Results:**

MND patients exhibited 18% higher whole-body mean muscle relative T2-signal than controls (95% CI 7–29%, *p* < 0.01), maximal in leg muscles (left tibialis anterior 71% (95% CI 33–122%, *p* < 0.01). Clinical and electrophysiological associations were evident. By 12 months, 16 patients had died or could not continue. In the remainder, relative T2-signal increased over 12 months by 14–29% in right tibialis anterior, right quadriceps, bilateral hamstrings and gastrocnemius/soleus (*p* < 0.01), independent of onset-site, and paralleled progressive weakness and electrophysiological loss of motor units. Highest clinical, electrophysiological and radiological SRMs were found for revised ALS-functional rating scale scores (1.22), tibialis anterior MUNIX (1.59), and relative T2-weighted leg muscle MR (right hamstrings: 0.98), respectively. Diffusion MR detected minimal changes.

**Conclusion:**

MUNIX and relative T2-weighted MR represent objective surrogate markers of progressive denervation in MND. Radiological changes were maximal in leg muscles, irrespective of clinical onset-site.

**Electronic supplementary material:**

The online version of this article (10.1007/s00415-019-09580-x) contains supplementary material, which is available to authorized users.

## Introduction

A significant challenge in motor neuron disease/amyotrophic lateral sclerosis (MND/ALS) research is the facility to track disease changes objectively over manageable time-scales, to reduce the duration and expense of clinical trials. Whilst survival remains a commonly applied outcome measure, slower progressing patients appear relatively over-represented in clinical trials [1, 2], and surrogate outcome measures are necessary to detect therapeutic effects in this group. The revised ALS functional rating scale (ALSFRS-R) questionnaire [3] is frequently used, but has well recognized limitations, including inherent subjectivity and influence of symptomatic treatment [4, 5]. Objective biomarkers are, therefore, required; imaging and electrophysiology appear promising candidates [6, 7].

Clinical heterogeneity in anatomical site of onset, pattern of spread, and rate of deterioration are important barriers to quantifying progression at group-level, whether using clinical, electrophysiological or radiological measures. Most previous imaging studies have focused on the central nervous system (116 in a recent review [8]), and there are relatively few studies of MND effects on peripheral nerve [9–11] or muscle [9, 10, 12–17], yet denervation and muscle weakness are cardinal clinico-pathological features. Approximately 25% of MND patients present with bulbar weakness and 70% with either upper or lower limb muscular weakness in similar proportions [18]. It is, therefore, challenging to capture the disparate effects of denervation on an individual’s muscles objectively and translate into a group-level parameter suitable for a trial. This may be addressed by application of clinical scores or electrophysiology to multiple muscles, or by whole-body muscle magnetic resonance (MR) imaging. In previous work, we reported longitudinal relative T2-weighted changes, derived from whole-body MR, in tibialis anterior over 4 months [17]. In this study, we present a new and comprehensive analysis of a wide range of clinical, electrophysiological and radiological muscle measures, including both T2- and diffusion-weighted MR, tested in multiple muscles over an extended follow-up period of 12 months. The aim was to identify individualized muscle denervation patterns in MND, and the objective was to assess the optimal technique to detect group-level change from a variety of clinical, electrophysiological and radiological candidates. We hypothesized that whole-body T2- and diffusion-weighted muscle MR would enable quantification of generalized denervation, regardless of clinical site of onset.

## Methods

### Study population

This was a prospective, longitudinal, observational cohort study. Patients were identified at first presentation to the tertiary referral neuromuscular clinic at the Royal Hallamshire Hospital, Sheffield, UK and were assessed at baseline, 4 and 12 months between October 2013 and May 2016. Inclusion criteria were age > 18 years, a clinical diagnosis of ALS fulfilling El Escorial criteria [19] or progressive muscular atrophy. Participation in interventional studies was recorded. Exclusion criteria were cognitive impairment sufficient to impair consent, contraindications to MR imaging, pregnancy, another neuromuscular disease, or respiratory failure impairing the ability to lie flat in the scanner. Healthy controls were recruited from partners of patients and by advertisement, and assessed at two time-points. Based on the results of our previous study [17], the primary outcome selected was between-group differences in relative T2-weighted MR signal over time and, in order to satisfy the requirement of at least 10–20 observations per degree of freedom for the linear regression models (with age and gender as covariates), a minimum sample size of 30–60 observations was required [20]. Secondary outcomes were between-group differences in clinical, electrophysiological and diffusion-weighted MR measures, inter-modality associations and change over time.

### Clinical assessments

Demographic data, site of onset and duration of weakness were recorded. At each visit, the following data were collected: weight; revised ALSFRS-R [3]; Medical Research Council scores [21] from bilateral deltoids, biceps brachii, triceps, wrist flexors, wrist extensors, finger flexors, finger extensors, abductor pollicis brevis, first dorsal interosseous, abductor digiti minimi, hip flexors, hip extensors, hamstrings, quadriceps, ankle dorsiflexors, ankle plantar flexors, and neck flexors and neck extensors, resulting in an MRC summary score (maximum 170); hand-held dynamometry scores in bilateral first dorsal interosseous, abductor pollicis brevis, abductor digiti minimi, quadriceps and ankle dorsiflexors.

### Electrophysiology

Compound muscle action potentials (CMAPs), motor unit number index (MUNIX) and motor unit size (MUSIX) [22] were obtained from the least clinically affected side, to avoid “floor” effects using surface electromyography (Dantec Keypoint, Natus Medical, California) following standardized protocols [23] in the following muscles: biceps brachii, abductor pollicis brevis, abductor digit minimi, tibialis anterior, abductor hallucis, and extensor digitorum brevis. In healthy controls, the right side was tested.

### Radiology

T2-weighted fast spin-echo and diffusion-weighted imaging (DWI) sequences were obtained at 3 T (Philips Ingenia, Best, Netherlands) with the following parameters: T2: TR = 1107 ms, TE = 80 ms, interpolated voxel size 0.78 × 0.78 × 5 mm^3^, 5–6 stations, 50 coronal slices, reformatted to correspond to axial DWI acquisitions; DWI: TR = 9412 ms, TE = 66 ms, TI = 250 ms, b0, b1000 s/mm^2^, voxel size 2.3 × 2.3 × 5 mm^3^, eight stations, 50 axial slices. Total acquisition times including localizers and breath-holds were approximately 20 min and 40 min for the T2- and diffusion-weighted acquisitions, respectively.

**Fig. 1:**
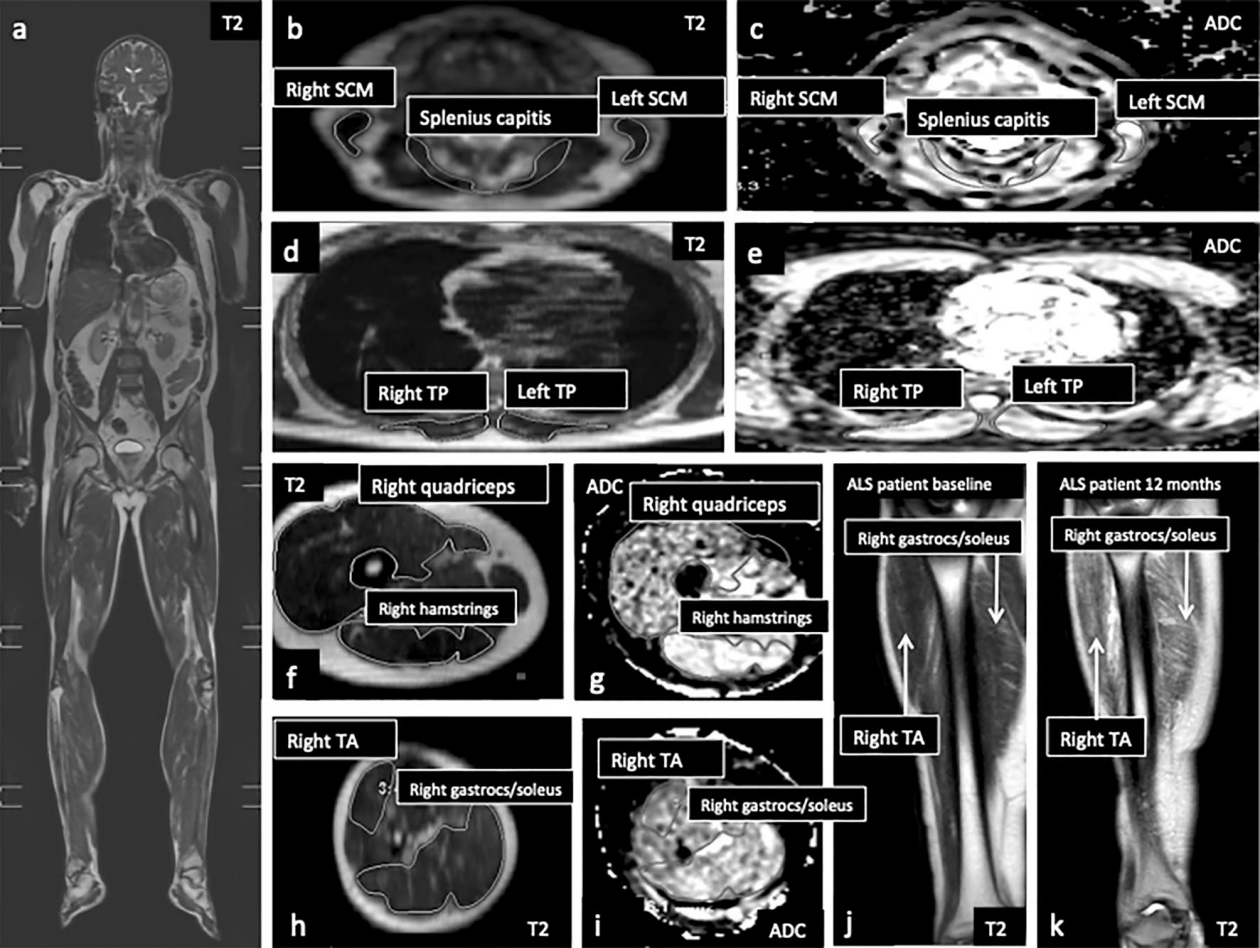
Coronal whole-body T2-weighted acquisition (**a**); axial slices from relative T2-weighted (**b**, **d**, **f**, **h**) and apparent diffusion coefficient (**c**, **e**, **g**, **i**) maps from head and neck station depicting right and left sternocleidomastoid and splenius capitis (**b**, **c**); thoracic station depicting right and left thoracic paraspinals (**d**, **e**); upper leg station depicting right quadriceps and hamstring groups (**f**, **g**) and lower leg station depicting right tibialis anterior and gastrocnemius/soleus groups (**h**, **i**). Coronal images from the lower leg station shown to illustrate an increase in relative T2-weighted signal in tibialis anterior and gastrocnemius/soleus groups in an MND patient between baseline (**j**) and 12 months (**k**). *ADC* apparent diffusion coefficient, *gastrocs* gastrocnemius, *SCM* sternocleidomastoid, *TA* tibialis anterior, *TP* thoracic paraspinal

Muscle regions-of-interest were contoured by two observers using standardized anatomical landmarks using a semi-automated spline function (Extended MR Workspace V2.6.3.5, Philips) on single slices for both T2- and diffusion-weighted images (Fig. 1a–i). Prior to analysis, intra- and inter-rater reproducibility was confirmed by coefficients of variability of < 5% for all regions-of-interest on six datasets reassessed after > 24 h. Mean relative T2 estimates were obtained from the following muscles and muscle groups in axial orientation: tongue, splenius capitis, bilateral trapezius, sternocleidomastoid, deltoid, biceps brachii, forearm compartment encompassing brachioradialis, thoracic paraspinal, psoas major, gluteus maximus, quadriceps, hamstrings, tibialis anterior, and gastrocnemius/soleus. Triceps, first dorsal interosseous, thenar and hypothenar eminence were also assessed but on coronal rather than axial T2 images, as anatomical boundaries were more consistently identifiable. Apparent diffusion coefficient (ADC) estimates were obtained from each of the muscles assessed in axial orientation and not from intrinsic hand muscles and triceps. To adjust for coil-loading effects, relative T2 estimates were expressed as a ratio to a bone reference within the same acquisition station [17] (Supplemental Material); no adjustment was made for ADC.

### Statistical analysis

Stata version 13.1 was used (StataCorp, Texas). For between-group comparisons and associations, p values were reported corrected for age and gender, due to potential influences on muscle parameters [24]. All *p* values were corrected for multiple comparisons by applying the Benjamini–Hochberg method to each table of results at each time-point, [25] and results where significance was retained were asterisked.

#### Baseline differences between MND patients and controls

For continuous variables, between-group differences were assessed using multiple regression models, entering each clinical, electrophysiological and radiological variable of interest, in turn, as the dependent variable, and group (patient/control), age and gender entered as independent variables. Between-group differences in categorical variables were assessed using chi-squared tests.

Results were reported as the difference in each parameter between patients and controls, derived from the regression models, expressed as a percentage ratio, with between-group difference the numerator, and control mean the denominator. Ratio 95% confidence intervals were calculated [26]. For ordinal MRC scores, the proportion of patients with weakness in each muscle (MRC < 5) was reported.

#### Clinical, electrophysiological and radiological associations

Associations between clinical, electrophysiological and radiological variables were assessed using separate multiple regression models, entering each clinical or electrophysiological variable, in turn, as the dependent variable, and the anatomically corresponding radiological variable, for relative T2 and ADC in each muscle, in turn, as an independent variable. Age and gender were entered into the model as additional independent variables.

#### Longitudinal changes

For continuous variables, longitudinal changes were modelled using mixed effects linear regression, with each clinical, electrophysiological and radiological variable entered, in turn, as the dependent variable, and time-point (as a categorical variable) and subject entered as independent variables. No assumptions were made on covariance structure. All available data were entered. Separate models were run for each variable, and for patients and controls. For radiological variables, percentage signal change compared to baseline was reported.

In addition to investigating each individual muscle separately, two additional analyses were performed to assess performance of radiological muscle estimates individualized to clinical onset-site to determine whether it was possible to increase sensitivity to detect group-level effects by individualizing damage measures to anatomical site of onset. First, for each subject, a single muscle was chosen to represent onset-site: tongue for bulbar-onset, right or left first dorsal interosseous for upper limb-onset and tibialis anterior for lower limb-onset (chosen because commonly clinically affected) [27]. These signal estimates were specified as a “muscle-of-onset” dependent variable, into a mixed effects regression model, entering time-point and subject as independent variables.

Second, for each subject, mean signal estimates were calculated from all muscles in the region-of-onset (tongue, trapezius and sternocleidomastoids for bulbar-onset; right or left deltoid, biceps, triceps, forearm compartment, first dorsal interosseous, thenar and hypothenar eminence for upper limb-onset; right or left psoas, gluteus maximus, quadriceps, hamstrings, tibialis anterior and gastrocnemius for lower limb-onset). These estimates were specified as a “region-of-onset” dependent variable, into a mixed effects regression model, entering time-point and subject as independent variables.

To compare these different strategies for detecting longitudinal relative T2-signal change in individuals, plots for each patient were reported for the following measures, selected post-hoc: whole-body muscle summary mean, region-of-onset, muscle-of-onset and a single leg muscle (right tibialis anterior).

The responsiveness of each normally distributed longitudinal outcome measure was reported using standardized response means (mean change between baseline and 12 months divided by its standard deviation); values > 0.8 are considered highly responsive [28].

To quantify within-subject heterogeneity for each measure, variance ratios were reported, derived from regression model outputs, by dividing the variance of the regression model constant (the fixed effects, representing group-level disease effect) by the summed variance of the constant and residual variance (the random effects, representing inter-individual variability). Lower values indicate greater relative within-group phenotypic variability.

Median differences in ordinal MRC scores were assessed using Wilcoxon matching-pairs tests.

#### Baseline predictors of muscle weakness

To determine whether baseline relative T2-weighted muscle signal predicted development of weakness at four and 12 months, clinical change variables were generated by calculating MRC score differences (four and 12 months minus baseline, respectively). Each of these change variables was entered as the dependent variable in separate regression models with baseline relative T2 from the corresponding muscle group as the independent variable. This analysis was performed only in muscles with corresponding clinical and radiological data, namely splenius capitis, deltoid, biceps brachii, first dorsal interossei, psoas major, gluteus maximus, quadriceps, hamstrings, tibialis anterior, and gastrocnemius/soleus. To determine whether relative T2-signal in clinically strong muscles was associated with development of weakness, the analysis was repeated after excluding muscles with MRC score < 5/5; sample sizes for each muscle are reported in Table 1.

**Table 1:** Radiological differences between MND patients and controls, and proportion of patients with clinical weakness, by muscle, at baseline, listed in order of maximal significant radiological differences

Muscle	Mean % difference in relative T2-signal patients > controls (95% CI)	*p* value	Proportion of patients with clinical weakness MRC < 5 (95% CI)
Left tibialis anterior	**71.4 (32.7, 122.2)**	< 0.001*	0.43 (0.26, 0.62)
Left quadriceps	**42.1 (14.0, 74.2)**	0.004*	0.04 (0.005, 0.23)
Right quadriceps	**37.9 (14.4, 63.8)**	0.002*	0.04 (0.005, 0.23)
Left gastrocnemius/soleus	**37.8 (11.7, 66.7)**	0.005*	0.14 (0.05, 0.33)
Right gastrocnemius/soleus	**37.6 (15.0, 62.4)**	0.002*	0.11 (0.03, 0.30)
Right hamstrings	**36.3 (7.4, 68.8)**	**0.015**	0.39 (0.23, 0.59)
Left hamstrings	**33.7 (9.3, 60.5)**	0.008*	0.36 (0.20, 0.55)
Right hypothenar eminence	**31.3 (4.0, 61.7)**	**0.028**	0.61 (0.41, 0.77)
Right tibialis anterior	**30.4 (5.5, 57.5)**	**0.018**	0.50 (0.32, 0.68)
Right thoracic paraspinals	**26.9 (2.3, 53.5)**	**0.033**	Not tested
Left hypothenar eminence	**26.5 (5.1, 49.3)**	**0.017**	0.82 (0.63, 0.93)
Left thoracic paraspinals	**26.1 (0.2, 54.0)**	**0.049**	Not tested
Right first dorsal interosseus	**24.7 (1.4, 49.7)**	**0.040**	0.64 (0.45, 0.80)
Right biceps brachii	**22.8 (7.5, 38.8)**	0.004*	0.21 (0.10, 0.41)
Summary mean	**18.0 (6.7, 29.5)**	0.001*	Not applicable
Right deltoid	**15.0 (0.8, 29.5)**	**0.039**	0.39 (0.23, 0.59)
Tongue	**11.4 (1.7, 21.2)**	**0.022**	Not tested
Left first dorsal interosseous	57.8 (− 2.8, 151.1)	0.062	0.68 (0.48, 0.83)
Left thenar eminence	24.1 (− 26.0, 82.0)	0.330	0.50 (0.32, 0.68)
Right thenar eminence	20.5 (− 1.8, 44.0)	0.073	0.50 (0.32, 0.68)
Left gluteus maximus	15.7 (− 34.1, 70.3)	0.517	0.04 (0.005, 0.23)
Left deltoid	12.6 (− 0.1, 25.7)	0.053	0.32 (0.17, 0.52)
Right gluteus maximus	12.1 (− 21.4, 47.3)	0.365	0.04 (0.005, 0.23)
Left biceps brachii	11.6 (− 0.2, 25.4)	0.094	0.11 (0.03, 0.30)
Left trapezius	10.4 (− 4.9, 26.1)	0.374	Not tested
Right triceps	10.1 (− 30.3, 52.3)	0.609	0.14 (0.05, 0.33)
Right sternocleidomastoid	8.7 (− 4.0, 21.8)	0.173	Not tested
Left sternocleidomastoid	7.9 (− 3.1, 19.0)	0.153	Not tested
Splenius capitis	5.2 (− 4.8, 15.2)	0.302	0.03 (0.004, 0.22)
Right psoas	4.6 (− 32.5, 42.4)	0.798	0.64 (0.45, 0.80)
Left forearm compartment	3.2 (− 27.1, 33.8)	0.828	Not tested
Left psoas	1.3 (− 46.1, 46.4)	0.995	0.64 (0.45, 0.80)
Right trapezius	0.6 (− 11.4, 11.5)	0.991	Not tested
Right forearm compartment	-8.9 (− 36.2, 17.7)	0.502	Not tested
Left triceps	− 20.0 (− 52.6, 10.5)	0.192	0.14 (0.03, 0.33)

Significant differences in radiological parameters between MND patients and controls are highlighted in bold. Results surviving multiple comparisons correction are asterisked

*CI* confidence interval, *MRC* Medical Research Council

## Results

### Study population

**Fig. 2:**
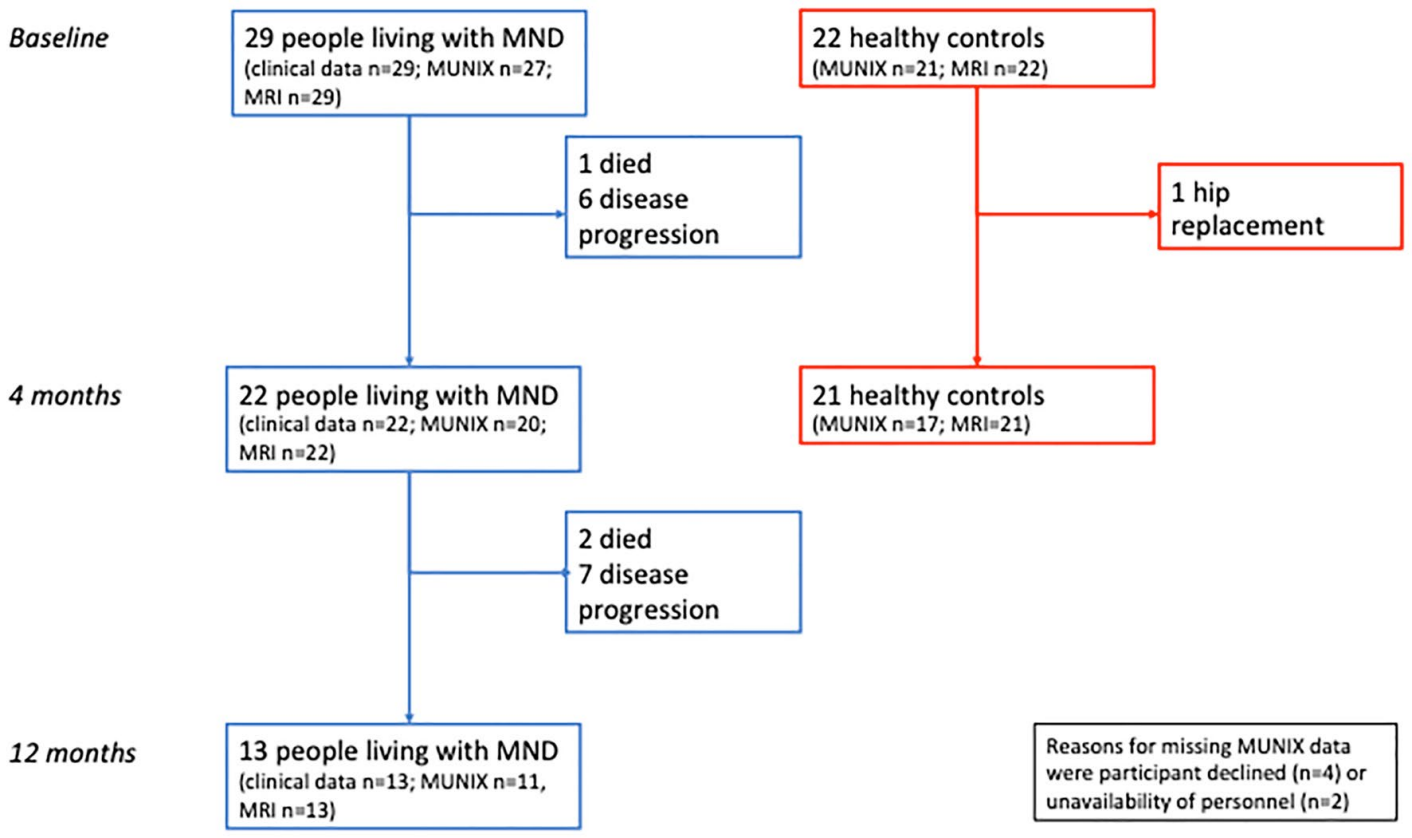
Participant follow-up. *MND* motor neuron disease, *MRI* magnetic resonance imaging, *MUNIX* motor unit number index

Twenty-nine MND patients (26 ALS and 3 progressive muscular atrophy) and 22 healthy volunteers entered the study. Follow-up rates are reported in Fig. 2. No patients participated in any interventional research during the course of the study. There were no differences in age, gender and weight between patients [mean age 57 years (SD 14), 7 females, mean weight 78 kg (SD 15)] and controls [54 years (SD 16), 9 females, 75 kg (SD 15), all *p* > 0.05]. In patients, mean ALSFRS-R and median MRC summary score were 40/48 (SD 4.5) and 161/170 (SD 10.6), respectively. Fourteen patients presented with upper limb-onset, 11 patients lower limb-onset, and four patients bulbar-onset disease. Patients were assessed at a median of 66 weeks from symptom onset (median 90 and 57 weeks for 12 month completer and non-completer subgroups, respectively). Median follow-up was at 19 and 55 weeks for patients and 29 weeks for controls.

### Baseline differences between MND patients and controls

Radiological and clinical differences are reported in Table 1. There were significant differences in relative T2 signal but no significant differences in apparent diffusion coefficient (ADC) between patients and controls. Electrophysiological differences are reported in Table 2.

**Table 2:** Neurophysiological differences between MND patients and controls, by muscle, at baseline, listed in order of maximal MUNIX differences

Muscle	Percentage differences between patients and controls (95% CI)					
	MUNIX	*p* value	MUSIX	*p* value	CMAP	*p* value
Abductor pollicis brevis	− **50 (**− **67, **− **34)**	< 0.001*	17 (− 4, 39)	0.115	− **37.7 (**− **55.3, **− **20.1)**	< 0.001*
Abductor hallucis	− **50 (**− **71, **− **29)**	< 0.001*	− 7 (− 33, 20)	0.611	− **41.5 (**− **61.1, **− **21.8)**	< 0.001*
Extensor digitorum brevis	− **44 (**− **69, **− **19)**	0.001*	− 21 (− 51, 10)	0.180	− **41.8 (**− **64.0, **− **19.6)**	< 0.001*
Tibialis anterior	− **39 (**− **56, **− **22)**	< 0.001*	**43 (11, 76)**	0.011*	− **22.2 (**− **40.2, **− **4.3)**	0.016*
Biceps brachii	− **36 (**− **52, **− **20)**	< 0.001*	4 (− 8, 16)	0.522	− **30.2 (**− **46.6, **− **13.9)**	0.001*
Abductor digiti minimi	− **34 (**− **51, **− **16)**	< 0.001*	20 (− 1, 41)	0.057	− **29.4 (**− **45.5, **− **15.3)**	< 0.001*

Significant differences between MND patients and controls are highlighted in bold. Results surviving multiple comparisons correction are asterisked. The least affected muscle in each patient and the right side in controls were tested

*CI* confidence interval, *CMAP* compound muscle action potential, *MUNIX* motor unit number index, *MUSIX* motor unit number size

### Clinical, electrophysiological and radiological associations

Associations between relative T2-weighted MR in each tested muscle with clinical power using hand-held dynamometry and MUNIX are reported in Table 3.

**Table 3:** Clinico-electrophysiological-radiological associations in MND patients

Muscle	Mean (SD) dynamometry (lb)	Mean (SD) MUNIX	Clinico-radiological association coefficient (95% CI)	*p* value	Electro-physiological-radiological association coefficient (95% CI)	*p* value
**Right biceps**	Not tested	**129.3 (53.9)**	Not tested	Not tested	9.7 (**− **128.9, 148.4)	0.885
**Left biceps**						
**Right first dorsal interosseous**	**4.8 (3.5)**	Not tested	**− 10.9 (− 17.0, − 4.8)**	0.001*	Not tested	Not tested
**Left first dorsal interosseous**	**4.7 (3.4)**		**− 6.6 (− 11.8, − 1.5)**	0.014*		
**Right abductor pollicis brevis**	**6.4 (4.6)**	**82.8 (52.2)**	**− 13.7 (− 24.5, − 2.9)**	0.015*	**− **74.2 (**− **207.1, 58.7)	0.258
Left abductor pollicis brevis	7.0 (5.1)		6.0 (**− **3.4, 15.4)	0.199		
Right abductor digiti minimi	3.4 (2.1)	123.2 (68.0)	**− **8.0 (**− **16.4, 0.4)	0.061	**− **86.9 (**− **380.9, 207.1)	0.543
**Left abductor digiti minimi**	**2.9 (1.9)**		**− 6.4 (− 12.2, − 0.6)**	**0.034**		
**Right quadriceps**	**42.8 (11.5)**	Not tested	**− 56.6 (− 84.8, − 28.4)**	< 0.001*	Not tested	Not tested
**Left quadriceps**	**43.6 (12.6)**		**− 51.2 (− 82.0, − 20.4)**	0.002*		
**Right tibialis anterior**	**34.0 (19.8)**	**83.5 (48.6)**	**− 93.0 (− 122.2, − 63.8)**	< 0.001*	**− 232.0 (− 343.1, − 120.9)**	< 0.001*
**Left tibialis anterior**	**36.5 (21.8)**		**− 108.1 (− 133.6, − 82.6)**	< 0.001*		

Significant associations between dynamometry scores, motor unit number index and relative T2-signal in corresponding muscles are reported in bold. Results surviving multiple comparisons correction are asterisked. Electrophysiological measures were derived from the least affected side in patients and regressed against clinical measures of corresponding laterality

*CI* confidence interval, *lb,* pounds force, *MUNIX* motor unit number index, *SD* standard deviation

For ADC, the only finding was that greater weakness in left tibialis anterior was associated with higher ADC (regression coefficient − 0.032 (− 0.057, − 0.008), *p* = 0.012*).

### Longitudinal changes

Longitudinal clinical, electrophysiological and relative T2-signal changes in MND patients are reported in Tables 4 and 5.

**Table 4:** Longitudinal clinical and electrophysiological changes in MND patients

Muscle	Mean difference between baseline and four months (95%CI)	*p* value	Mean difference between baseline and 12 months (95%CI)	*p* value	SRM	Var ratio
**Clinical scores**						
** ALSFRS-R**	**− 3.4 (− 4.9, − 1.8)**	< 0.001*	**− 5.7 (− 7.6, − 3.8)**	< 0.001*	**− 1.22**	**0.78**
**Dynamometry (lb)**						
** Right first dorsal interosseous**	**− 1.1− (− 1.9, − 0.4)**	0.005*	**− 1.9 (− 2.8, − 0.9)**	< 0.001*	**− 0.74**	**0.86**
** Left first dorsal interosseous**	**− 1.0 (− 1.7, − 0.3)**	0.004*	**− 2.0 (− 2.8, − 1.1)**	< 0.001*	**− 0.85**	**0.89**
Right abductor pollicis brevis	**− **0.2 (**− **1.5, 1.2)	0.804	**− **1.5 (**− **3.2, 0.2)	0.081	**− **0.38	0.79
Left abductor pollicis brevis	**− **1.1 (**− **2.9, 0.7)	0.250	**− **2.1 (**− **4.3, 0.2)	0.068	**− **0.43	0.69
** Right abductor digiti minimi**	**− **0.6 (**− **1.3, 0.1)	0.070	**− 1.0 (− 1.8, − 0.2)**	0.015*	**− 0.36**	**0.70**
** Left abductor digiti minimi**	**− 0.8 (− 1.3, − 0.2)**	0.005*	**− 1.4 (− 2.0, − 0.7)**	< 0.001*	**− 0.89**	**0.79**
Right quadriceps	**− **3.1 (**− **7.4, 1.1)	0.149	**− **4.6 (**− **9.8, 0.6)	0.085	**− **0.46	0.63
Left quadriceps	**− **2.8 (**− **7.6, 2.0)	0.250	**− **3.6 (**− **9.5, 2.3)	0.234	**− **0.36	0.57
** Right tibialis anterior**	**− **4.9 (**− **10.0, 0.3)	0.064	**− 10.7 (− 17.0, − 4.4)**	0.001*	**− 0.73**	**0.85**
** Left tibialis anterior**	**− 8.4 (− 14.6, − 2.2)**	0.008*	**− 9.3 (− 16.9, − 1.7)**	0.016*	**− 0.64**	**0.79**
**MUNIX**						
** Biceps brachii**	**− 19 (− 33, − 4)**	0.013*	**− 23 (− 42, − 4)**	0.017*	**− 0.45**	**0.85**
** Abductor pollicis brevis**	**− 23 (− 33, − 12)**	< 0.001*	**− 33 (− 46, − 20)**	< 0.001*	**− 1.47**	**0.90**
** Abductor digiti minimi**	**− 45 (− 62, − 21)**	< 0.001*	**− 66 (− 92, − 41)**	< 0.001*	**− 1.08**	**0.72**
** Tibialis anterior**	**− 11 (− 17, − 5)**	< 0.001*	**− 20 (− 27, − 13)**	< 0.001*	**− 1.59**	**0.96**
** Abductor hallucis**	**− **1 (**− **23, 6)	0.234	**− 28 (− 45, − 11)**	0.001*	**− 0.72**	**0.93**
Extensor digitorum brevis	**− **2 (**− **12, 8)	0.645	**− **10 (**− **23, 2)	0.108	**− **0.40	0.83
**MUSIX**						
Biceps brachii	2 (**− **1, 6)	0.212	1 (**− **3, 6)	0.710	0.17	0.67
Abductor pollicis brevis	5 (**− **22, 33)	0.701	20 (**− **14, 53)	0.253	0.23	0.21
** Abductor digiti minimi**	6 (**− **9, 21)	0.448	**33 (15, 51)**	< 0.001*	**1.04**	**0.27**
Tibialis anterior	5 (**− **12, 22)	0.537	**− **3 (**− **23, 17)	0.778	**− **0.12	0.68
** Abductor hallucis**	3 (**− **3, 9)	0.317	**8 (1, 15)**	0.031*	**0.92**	**0.92**
Extensor digitorum brevis	1 (**− **14, 16)	0.852	5 (**− **14, 23)	0.621	0.05	0.83
**CMAP**						
** Biceps brachii**	**− 0.7 (− 1.2, − 0.2)**	0.004*	**− 1.2 (− 1.8 to − 0.5)**	0.001*	**− 0.86**	**0.88**
** Abductor pollicis brevis**	**− 1.1 (− 1.6, − 0.6)**	< 0.001*	**− 2.1 (− 2.8 to − 1.5)**	< 0.001*	**− 1.36**	**0.93**
** Abductor digiti minimi**	**− 1.2 (− 2.0, − 0.4)**	0.002*	**− 2.2 (− 3.1 to − 1.2)**	< 0.001*	**− 0.87**	**0.86**
** Tibialis anterior**	**− **0.3 (**− **0.7, 0.1)	0.191	**− 1.0 (− 1.5, − 0.5)**	< 0.001*	**− 0.85**	**0.89**
** Abductor hallucis**	**− **0.6 (**− **1.2, 0.1)	0.099	**− 1.2 (− 2.0, − 0.4)**	0.003*	**− 0.66**	**0.95**
** Extensor digitorum brevis**	**− **0.1 (**− **0.6, 0.3)	0.553	**− 0.7 (− 1.3, − 0.1)**	0.017*	**− 0.86**	**0.92**

Significant changes in clinical and electrophysiological parameters in MND patients between baseline and subsequent four and 12 month time-points are highlighted in bold. Results surviving multiple comparisons correction are asterisked. Electrophysiological measures were derived from the least affected side in each patient at baseline and the same muscle was retested at each follow-up

*ALSFRS-R* amyotrophic lateral sclerosis functional rating scale- revised*, CI* confidence interval, *CMAP* compound muscle action potential, *lb* pounds force, *MUNIX* motor unit number index, *MUSIX* motor unit number size, *SRM* standardized response mean, *Var* variance

**Table 5:** Longitudinal radiological changes in relative T2-signal in MND patients

Muscle	Regression coefficientMean % change in relative T2-signal between baseline and four months (95%CI)	*p* value	Regression coefficientMean % change in relative T2-signal between baseline and 12 months (95%CI)	*p* value	SRM	Var ratio
Tongue	**− **0.04 (**− **0.09, 0.01)** − **4.8 (**− **11.2, 1.5)	0.123	**− **2.4–0.02 (**− **10.20.08, 5.4 0.04)	0.540	**− **0.08	0.66
Right trapezius	**− **6.90.03 (**− **19.10.09, 5.20.02)	0.251	0.20.001 (**− **14.50.07, 14.80.07)	0.981	0.06	0.17
Left trapezius	**− **10.90.06 (**− **22.90.1, 0.0904)	0.066	**− **1.7–0.009 (**− **16.10.08, 12.80.06)	0.816	**− **0.02	0.17
Right sternocleidomastoid	**− **3.70.02 (**− **13.40.06, 6.00.03)	0.443	0.3002 (**− **11.50.05, 12.20.05)	0.949	0.10	0.43
Left sternocleidomastoid	**− **4.40.03 (**− **14.50.08, 5.60.03)	0.376	**− **4.1 0.02 (**− **16.30.09, 8.20.04)	0.502	**− **0.11	0.37
Splenius capitis	4.20.03 (**− **4.30.03, 12.80.08)	0.321	**− **5.40.03 (**− **16.00.1, 5.10.03)	0.297	**− **0.42	0.47
Right deltoid	2.70.009 (**− **5.00.02, 10.40.04)	0.486	**− **4.80.02 (**− **14.40.05, 4.70.02)	0.304	**− **0.27	0.71
Left deltoid	1.90.008 (**− **6.60.03, 10.40.04)	0.654	4.60.02 (**− **5.80.02, 15.20.06)	0.365	0.08	0.59
Right biceps brachii	**− **2.20.01 (**− **13.80.06, 9.40.04)	0.700	11.3 0.05 (**− **2.9–0.01, 25.70.1)	0.107	0.18	0.55
Left biceps brachii	**− **3.5–0.009 (**− **17.40.05, 0.10.103)	0.597	**− **1.90.005 (**− **18.60.05, 14.70.04)	0.814	**− **0.21	0.26
Right triceps	**− **13.90.06 (**− **47.20.2, 18.00.07)	0.378	**− **22.30.09 (**− **65.10.2, 18.40.07)	0.266	**− **0.19	0.10
Left triceps	**− **0.00 (**− **31.3–0.08, 31.2 0.08)	0.998	–0.00.02 (**− **22.10.1, 22.10.09)	0.708	**− **0.18	0.00
Right brachioradialis	7.30.02 (**− **15.00.05, 29.0.09)	0.507	10.40.03 (**− **15.40.05, 36.80.1)	0.414	0.04	0.06
Left brachioradialis	7.00.02 (**− **16.80.05, 31.20.09)	0.551	9.90.03 (**− **19.30.06, 39.60.1)	0.492	0.82	0.23
**Right first dorsal interosseous**	18.4 0.1 (**− **1.8–0.007, 39.80.2)	0.067	**31.10.2 (6.80.04, 57.40.3)**	**0.010**	**0.64**	**0.43**
Left first dorsal interosseous	**− **0.002 (**− **26.20.07, 24.60.07)	0.952	16.10.05 (**− **16.20.05, 50.40.1)	0.311	0.97	0.41
Right thenar	14.40.07 (**− **7.10.03, 36.60.17)	0.177	21.40.1 (**− **4.90.02, 48.90.2)	0.100	0.76	0.26
Left thenar	**− **8.70.02 (**− **42.10.1, 23.80.06)	0.586	**− **10.0–0.03 (**− **50.10.1, 29.8 0.08)	0.607	**− **0.57	0.17
Right hypothenar	5.50.02 (**− **10.60.03, 22.00.06)	0.491	13.80.04 (**− **5.8, 0.02,34.4 0.1)	0.155	0.27	0.73
Left hypothenar	3.10.01 (**− **12.60.05, 18.80.08)	0.691	2.30.009 (**− **16.20.06, 20.90.08)	0.802	0.22	0.34
Right thoracic paraspinal	**− **1.00.005 (**− **4.40.09, 2.40.08)	0.903	16.70.09 (**− **3.00.01, 37.60.2)	0.087	0.30	0.69
Left thoracic paraspinal	1.60.01 (**− **12.10.08, 15.50.1)	0.811	4.20.03 (**− **12.70.08, 21.40.14)	0.610	0.24	0.70
**Right psoas**	6.20.03 (**− **19.90.08, 33.10.1)	0.627	**35.70.2 (4.60.02, 71.10.3)**	**0.020**	**0.53**	**0.52**
Left psoas	**− **11.80.04 (**− **42.80.1, 17.90.06)	0.419	**− **15.70.06 (**− **54.10.2, 20.90.07)	0.382	**− **0.01	0.31
Right gluteus maximus	**− **3.00.02 (**− **24.30.1, 18.10.1)	0.774	22.00.1 (**− **3.1–0.02, 48.80.3)	0.076	0.44	0.49
Left gluteus maximus	**− **14.2 0.1 (**− **42.30.3, 12.20.09)	0.277	–14.80.1 (**− **49.8–0.4, 18.40.1)	0.363	**− **0.10	0.53
**Right quadriceps**	5.40.02 (**− **2.5**− **,0.008, 13.60.05)	0.168	**018.8.07 (9.00.03, 29.60.1)**	< 0.001*	**0.86**	**0.90**
**Left quadriceps**	1.20.004 (**− **7.40.02, 9.70.03)	0.784	**11.40.04 (2.90.004, 20.60.07)**	**0.028**	**0.39**	**0.91**
**Right hamstrings**	**− **2.20.007 (**− **10.20.03, 5.60.02)	0.564	**17.40.05 (7.70.02, 28.20.08)**	< 0.001*	**0.98**	**0.93**
**Left hamstrings**	1.90.008 (**− **7.10.03, 11.00.05)	0.666	**14.40.06 (3.30.02, 26.30.1)**	0.008*	**0.65**	**0.89**
**Right tibialis anterior**	**10.70.04 (0.8004, 21.20.07)**	**0.030**	**28.50.1 (15.90.06, 42.70.1)**	< 0.001*	**0.82**	**0.87**
**Left tibialis anterior**	4.10.02 (**− **7.90.01, 16.50.06)	0.180	**20.90.07 (6.00.02, 37.20.11)**	0.004*	**0.33**	**0.86**
**Right gastrocnemius/soleus**	5.60.02 (**− **3.20.01, 14.70.06)	0.200	**18.90.07 (8.00.03, 30.60.1)**	< 0.001*	**0.64**	**0.83**
**Left gastrocnemius/soleus**	0.5003 (**− **8.20.04, 9.10.05)	0.911	**18.70.1 (8.0(0.05, 30.30.2)**	< 0.001*	**0.78**	**0.88**
Muscle**-**of**-**onset	**− **0.102 (**− **16.10.10, 16.0 0.07)	0.698	16.60.10 (**− **2.90.008, 37.3) 0.20)	0.070	0.77	0.73
**Region-of-onset**	2.70.01 (**− **4.30.02, 9.70.04)	0.437	**14.10.06 (5.50.03, 23.2 0.10)**	0.001*	**0.87**	**0.89**
**Summary mean**	**− **0.1.0004 (**− **6.20.03, 5.90.03)	0.972	**8.80.04 (1.30.007, 16.4 0.07)**	**0.017**	**0.36**	**0.56**

Significant T2-weighted radiological changes in MND patients between baseline and subsequent four and 12 month time-points are highlighted in bold. Results surviving multiple comparisons correction are asterisked

*CI* confidence interval*, SRM* standardized response mean, *Var* variance

Decreases in median MRC of up to one point were found in the following muscles at 4 months: left abductor pollicis brevis, right first dorsal interosseous, left wrist extensors, bilateral hamstrings, and right tibialis anterior; and in the following muscles at 12 months: left abductor pollicis brevis, bilateral first dorsal interossei, and left abductor digit minimi. Right tibialis anterior MRC decreased by 1.5 points at 12 months (all *p* < 0.05). Median MRC summary score was 103/170 at baseline, 94/170 at 4 months (*p* < 0.05), and 97/170 at 12 months (*p* < 0.05 from baseline).

ADC decreased only in patients’ right sternocleidomastoid at 12 months (regression coefficient − 366.1 (− 557.8, − 174.4), *p* < 0.001*).

There were no significant changes in healthy controls in any measure.

**Fig. 3:**
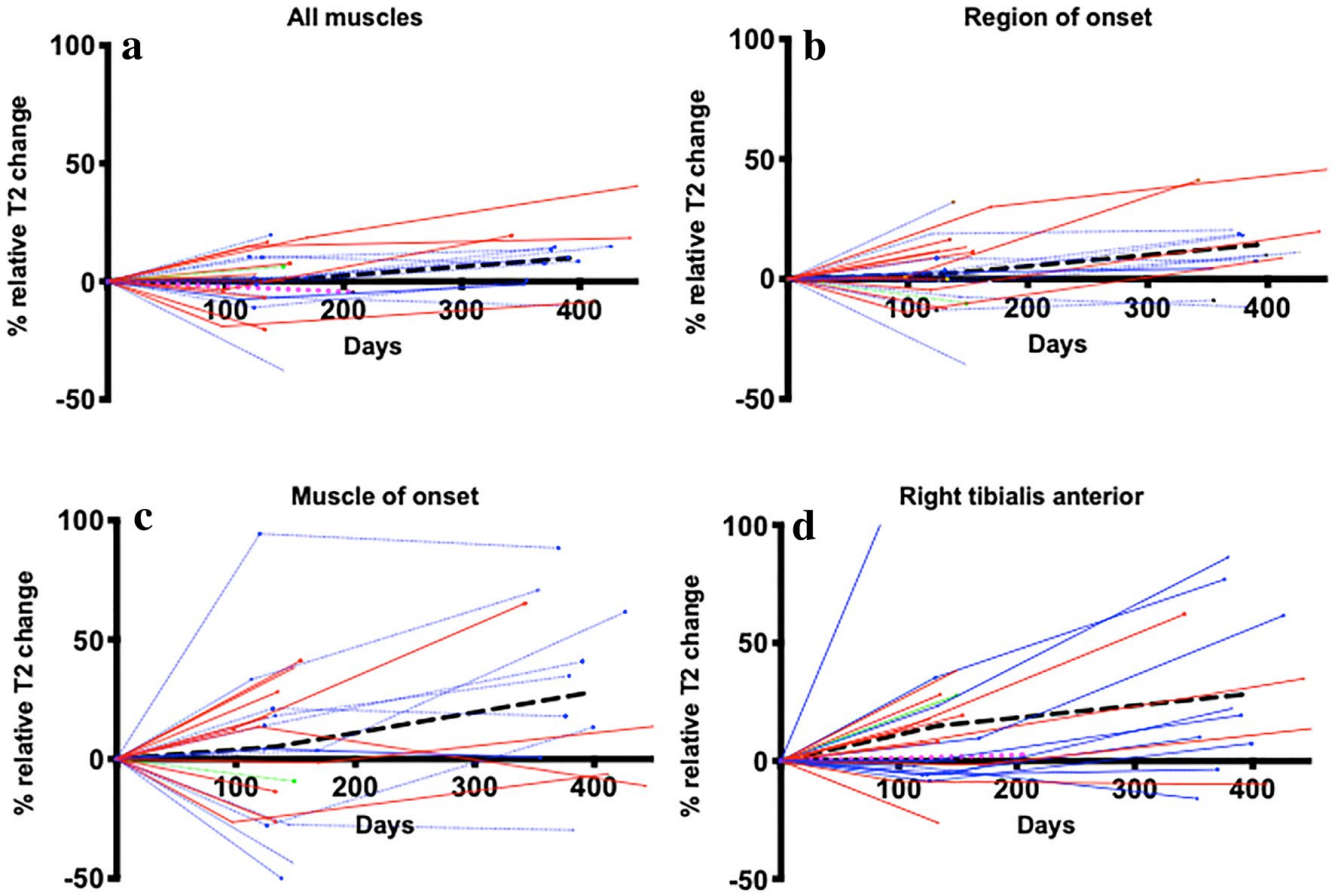
Longitudinal changes in MND patients in relative T2-weighted MR signal: whole-body summary mean (**a**); region-of-onset (**b**); single muscle-of-onset (**c**) and right tibialis anterior (**d**). Red lines indicate patients with lower limb-onset, blue lines upper limb-onset and green lines bulbar-onset. The bold black dashed line indicates all patients mean. The magenta dashed lines indicate the healthy control mean where relevant

Individualized plots of longitudinal relative T2-weighted changes summarized for all muscles, by region-of-onset, by muscle-of-onset and for a single leg muscle (right tibialis anterior), are illustrated in Fig. 3.

### Baseline predictors of subsequent muscle weakness

Associations between higher baseline relative T2 muscle signal and subsequent development of clinical weakness were found in right thenar eminence [regression coefficient (*r*) = − 3.88 (95% CI − 7.21, − 0.55), *p* = 0.025), right gluteus maximus (*r* = − 0.53 (− 0.93, − 0.12), *p* = 0.014), bilateral quadriceps (right: *r* = − 1.29 (− 2.53, − 0.04), *p* = 0.044; left: *r* = − 1.11 (− 2.00, − 0.21, *p* = 0.018) right hamstrings (*r* = − 1.63 (− 3.21, − 0.04), *p* = 0.045) and left gastrocnemius/soleus at four months (*r* = − 2.01 (− 2.93, − 1.08), *p* < 0.001*); and right hamstrings (*r* = − 2.24 (− 4.15, − 0.33), *p* = 0.025), right tibialis anterior (*r* = − 7.41 (− 12.47, − 2.35), *p* = 0.008) and left gastrocnemius/soleus at 12 months (*r* = − 2.89 (− 4.24, − 1.54), *p* = 0.001*).

When only clinically normal muscles at baseline were included, higher baseline relative T2-signal was associated with development of weakness in right gluteal muscles (*r* = − 0.72 (95% CI − 1.15, − 0.29), *p* = 0.002*), right hamstrings (*r* = − 2.00 (95% CI − 3.03, − 0.97), *p* = 0.001*) and left gastrocnemius (*r* = − 1.19 (95% CI − 2.00, − 0.38), *p* = 0.007*) at 4, but not 12 months.

## Discussion

This study represents the most comprehensive longitudinal analysis of muscle-based clinical, electrophysiological and imaging biomarkers in MND to date, combining multi-modal assessments across multiple muscles. The key result is that no single technique or muscle fully captured change at group level; different assessment tools were differentially sensitive in different muscles. We hypothesized that whole-body muscle imaging would capture widespread progression of denervation, but instead found that leg muscle changes were the most effective radiological biomarker in this cohort, regardless of clinical onset-site and, importantly, detected changes in slow progressors, an area of need for clinical trials.

At baseline, clinical weakness was frequent in left abductor digiti minimi (ADM), bilateral first dorsal interosseous and bilateral psoas. Of these muscles, ADM weakness is perhaps surprising, because generally considered relatively spared in MND, at least in terms of wasting (the basis of the split hand phenomenon), whilst involvement of first dorsal interosseous is typical [29]. Patients exhibited greater motor unit loss in abductor pollicis brevis than ADM at baseline, but MUNIX also dropped significantly in ADM over time, and this muscle appeared commonly affected in this cohort. In general, radiological changes were associated with clinical weakness more frequently than with electrophysiological motor unit loss, although associations with both were evident in tibialis anterior. Radiological increases in relative T2-signal likely reflect muscle fluid changes, and later fatty replacement [30], and appear a consistent finding in MND. Qualitative T2 changes have been reported in the tongue [12] and arm muscles [9, 10], and quantitative changes in a small cohort in leg muscles [13]. In a very recently published paper, differences between MND patients and healthy controls were demonstrated in leg muscles on T2-weighted short tau inversion recovery imaging evaluated with rater scales, but there were no differences in quantitative fat fraction imaging in either the leg muscles or tongue [31]. In contrast to T2 signal, muscle volume changes appear modest [14, 16, 17]. Our data suggest that muscle relative T2-signal change may capture aspects of pathophysiology contributing to weakness other than loss of electrophysiological motor units. Associations between high baseline relative T2-signal and development of weakness in some muscles, even when clinically strong, suggests this may occur early, an intriguing finding that merits further investigation.

The difficulties of capturing change in MND with simple clinical measures, such as MRC scores, were illustrated in this study and highlight the challenges of phenotypical heterogeneity. Group-level longitudinal changes were detectable in first dorsal interosseous and tibialis anterior on dynamometry, muscles generally recognized as typically affected in MND [29, 32], but this test is effort-dependent [4]; despite its known limitations, ALSFRS-R proved the most responsive longitudinal clinical measure in this study. This is likely to reflect the generally lower variance of ALSFRS-R compared to muscle T2 values outside the leg muscles, as illustrated in Tables 4 and 5, and the mortality-related attrition common to MND studies may also have biased the 12-month SRM estimate for ALSFRS-R. Muscle MR has some advantages over ALSFRS-R not captured by SRM estimates, namely objectivity, independence from potential confounds of therapeutic intervention, and assessment of pathophysiological effects rather than their symptomatic consequences. These assessment methods appear complementary. It is possible that a fully quantitative T2 relaxometry protocol could reduce the error variance and increase the responsiveness of the MR measurements, but this question cannot be answered by the present study.

On objective tests, progressive electrophysiological motor unit loss was evident, as in previous studies [33], especially in tibialis anterior and abductor pollicis brevis. Interestingly, there was only limited evidence of reinnervation on MUSIX, at baseline or longitudinally. We examined the strongest side in patients, which may indicate that MUSIX changes lag behind MUNIX, because subclinical or early motor unit loss had not yet triggered reinnervation. We also pooled weak and strong muscles which may have diluted overall differences in a relatively small cohort. Limitations of MUNIX/MUSIX are that patient effort is required, not all muscles are amenable to study, only relatively few can be assessed in a session, and “floor effects” exist. Our data suggest that tibialis anterior and abductor pollicis brevis represent good targets. Floor effects also exist for clinical measures, such as dynamometry and MRC scores. We did not adjust for this effect in our analysis (for example, by excluding patients with low MRC scores at baseline from further analysis). This could be explored in a larger, adequately powered cohort.

This was the first application of whole-body diffusion-weighted MR to assess muscle tissue integrity in MND. Very few changes were found, either because opposing effects of pathophysiological processes occurred or due to technical factors. It is possible that concurrent effects of myofibrillar cell membrane damage and increased intramuscular fluid increased diffusion, whilst consequent cellular debris and increased fat deposition caused a decrease, resulting in no detectable net ADC change. Alternatively, exponential signal intensity decay at high b values may have resulted in loss of signal. A previous study of muscle denervation in rats applied a lower b value of 600 s/mm^2^ [34], compared to *b* = 1000 s/mm^2^ used in this study. We conclude that T2-weighted muscle imaging approaches appear more sensitive to MND change than diffusion-weighted MR, at least using the parameters applied.

Leg muscles appear the best target for future fully quantitative T2 studies, although assessment in an independent cohort is necessary to determine whether this finding is generalizable. Whilst an increase in whole-body relative T2 was evident, this did not survive adjustment for multiple comparisons. Longitudinal changes were more readily detectable in the lumbar region, compared with cranial, cervical and thoracic body segments. This does not appear to be attributable to clinical factors; whilst lower limb-onset and progression were quite prevalent in our cohort, this was also the case for arm muscles. Technical factors may have contributed; leg muscles are larger, central within the acquisition field-of-view, with clearly defined anatomical boundaries, and these factors could influence the observed lower regression variance ratios. Measurement error might be reduced by developing fully automated analysis algorithms for whole-body MR in the future. Technical factors also prevented assessment of other muscles of interest, such as the diaphragm, which was not consistently identifiable using the slice thickness applied in this study. Thinner slices are possible but would necessitate longer scan-times.

Despite cohort attrition, typical of longitudinal cohort studies in MND, resulting in lower statistical power, longitudinal relative T2 changes from baseline were more marked at 12 than 4 months. In our previous study, which assessed this cohort to four months using different methodology, longitudinal changes were identified in tibialis anterior (and not in biceps brachii, thoracic paraspinals or the tongue) [17]. In the present analysis, similar results were found, despite a different methodology (assessing axial rather than coronal slices) performed by a different operator. Progressive denervation effects were again only found in leg muscles, including right tibialis anterior at both 4 and 12 months. Although the previously identified increase in relative T2 signal in left tibialis anterior did not reach statistical significance at 4 months in the present analysis (probably due to sampling differences), changes in this muscle were detectable at 12 months. Changes in dynamometry and electrophysiology were again evident in leg muscles. It is interesting to consider whether an MR “floor effect” exists, as for dynamometry and electrophysiology, when no further change is detectable because of complete paresis with absent motor potentials. This would require subgroup assessment in an adequately powered cohort.

A limitation of relative T2-weighted MR is the necessity to adjust measurements to reference tissue within each acquisition station to allow for differential coil-loading effects between participants, because the sequence is not fully quantitative. This could have biased between-muscle comparisons. We sought to minimize bias by reporting percentage T2-signal differences relative to healthy controls. Previous studies using similar sequences have applied qualitative grading scales and expert raters [9, 10]. We argue that our approach reduces subjectivity and has the advantage of producing continuous data, but measurement variance will be higher than fully quantitative T2 techniques. Despite these potential limitations, a clear pattern of biologically and clinically feasible results was evident. These considerations illustrate the necessary trade-off between the number of muscles that can be studied concurrently and a feasible scan-time for disabled MND patients. For similar reasons, we could not collect corresponding clinico-electrophysiological data for all muscles investigated with MR, or combine our assessments with other promising muscle techniques, such as electrical impedance myography [35]. Nevertheless, our dataset still represents the most wide-ranging imaging and electrophysiological muscle assessment in MND to date. Our cohort demonstrated the heterogeneity in disease progression rates typical of the ALS population. It would be interesting to assess the utility of muscle biomarkers in a cohort of ALS patients selected for slow progression (> 0.9 ALSFRS-R points/month), where these measures would add most value, in a future study.

In summary, this longitudinal study is the first to demonstrate clinically and electrophysiologically relevant progressive muscle denervation on MR across a wide range of muscle groups over 12 months. Although we hypothesized that whole-body muscle MR would capture generalized changes, our data suggest that leg muscles are sensitive to detect group-level longitudinal changes, irrespective of clinical onset-site, and could represent a biomarker target for future quantitative studies. Relative T2-weighted MR appeared more sensitive to detect denervation than diffusion-weighted MR.

## Electronic supplementary material

Below is the link to the electronic supplementary material.

Supplementary file1 (DOCX 107 kb)
