# Correction to: Longitudinal multi-modal muscle-based biomarker assessment in motor neuron disease

**DOI:** 10.1007/s00415-019-09648-8

**Published:** 2019-11-28

**Authors:** Thomas M. Jenkins, James J. P. Alix, Jacob Fingret, Taniya Esmail, Nigel Hoggard, Kathleen Baster, Christopher J. McDermott, Iain D. Wilkinson, Pamela J. Shaw

**Affiliations:** 1 Sheffield Institute for Translational Neuroscience, University of Sheffield, 385a Glossop Road, Sheffield, S10 2HQ UK; 2 Department of Neurology, Sheffield Teaching Hospitals NHS Foundation Trust, Sheffield, UK; 3 Departments of Neurophysiology, Sheffield Teaching Hospitals NHS Foundation Trust, Sheffield, UK; 4 Academic Unit of Radiology, University of Sheffield, Sheffield, UK; 5 Statistics Services Unit, School of Mathematics and Statistics, University of Sheffield, Sheffield, UK

## Correction to: Journal of Neurology 10.1007/s00415-019-09580-x

The original version of this article unfortunately contained a mistake. The numbers in the Table 5 appear to have been reproduced wrongly as dates, rather than percentages.

The corrected Table 5 is given in the next page.

**Table 5:** Longitudinal radiological changes in relative T2-signal in MND patients

Muscle	Mean % change in relative T2-signal between baseline and 4 months (95% CI)	*P* value	Mean % change in relative T2-signal between baseline and 12 months (95% CI)	*P* value	SRM	Var ratio
Tongue	− 4.8 (− 11.2, 1.5)	0.123	− 2.4 (− 10.2, 5.4)	0.540	− 0.08	0.66
Right trapezius	− 6.9 (− 19.1, 5.2)	0.251	0.2 (− 14.5, 14.8)	0.981	0.06	0.17
Left trapezius	− 10.9 (− 22.9, 0.9)	0.066	− 1.7 (− 16.1, 12.8)	0.816	− 0.02	0.17
Right sternocleidomastoid	− 3.7 (− 13.4, 6.0)	0.443	0.3 (− 11.5, 12.2)	0.949	0.10	0.43
Left sternocleidomastoid	− 4.4 (− 14.5, 5.6)	0.376	− 4.1 (− 16.3, 8.2)	0.502	− 0.11	0.37
Splenius capitis	4.2 (− 4.3, 12.8)	0.321	− 5.4 (− 16.0, 5.1)	0.297	− 0.42	0.47
Right deltoid	2.7 (− 5.0, 10.4)	0.486	− 4.8 (− 14.4, 4.7)	0.304	− 0.27	0.71
Left deltoid	1.9 (− 6.6, 10.4)	0.654	4.6 (− 5.8, 15.2)	0.365	0.08	0.59
Right biceps brachii	− 2.2 (− 13.8, 9.4)	0.700	11.3 (− 2.9, 25.7)	0.107	0.18	0.55
Left biceps brachii	− 3.5 (− 17.4, 10.1)	0.597	− 1.9 (− 18.6, 14.7)	0.814	− 0.21	0.26
Right triceps	− 13.9 (− 47.2, 18.0)	0.378	− 22.3 (− 65.1, 18.4)	0.266	− 0.19	0.10
Left triceps	− 0.0 (− 31.3, 31.2)	0.998	− 0.0 (− 22.1, 22.1)	0.708	− 0.18	0.00
Right brachioradialis	7.3 (− 15.0, 29.9)	0.507	10.4 (− 15.4, 36.8)	0.414	0.04	0.06
Left brachioradialis	7.0 (− 16.8, 31.2)	0.551	9.9 (− 19.3, 39.6)	0.492	0.82	0.23
**Right first dorsal interosseous**	18.4 (− 1.8, 39.8)	0.067	**31.1 (6.8, 57.4)**	**0.010**	**0.64**	**0.43**
Left first dorsal interosseous	− 0.0 (− 26.2, 24.6)	0.952	16.1 (− 16.2, 50.4)	0.311	0.97	0.41
Right thenar	14.4 (− 7.1, 36.6)	0.177	21.4 (− 4.9, 48.9)	0.100	0.76	0.26
Left thenar	− 8.7 (− 42.1, 23.8)	0.586	− 10.0 (− 50.1, 29.8)	0.607	− 0.57	0.17
Right hypothenar	5.5 (− 10.6, 22.0)	0.491	13.8 (− 5.8, 34.4)	0.155	0.27	0.73
Left hypothenar	3.1 (− 12.6, 18.8)	0.691	2.3 (− 16.2, 20.9)	0.802	0.22	0.34
Right thoracic paraspinal	− 1.0 (− 4.4, 2.4)	0.903	16.7 (− 3.0, 37.6)	0.087	0.30	0.69
Left thoracic paraspinal	1.6 (− 12.1, 15.5)	0.811	4.2 (− 12.7, 21.4)	0.610	0.24	0.70
**Right psoas**	6.2 (− 19.9, 33.1)	0.627	**35.7 (4.6, 71.1)**	**0.020**	**0.53**	**0.52**
Left psoas	− 11.8 (− 42.8, 17.9)	0.419	− 15.7 (− 54.1, 20.9)	0.382	− 0.01	0.31
Right gluteus maximus	− 3.0 (− 24.3, 18.1)	0.774	22.0 (− 3.1, 48.8)	0.076	0.44	0.49
Left gluteus maximus	− 14.2 (− 42.3, 12.2)	0.277	− 14.8 (− 49.8, 18.4)	0.363	− 0.10	0.53
**Right quadriceps**	5.4 (− 2.5, 13.6)	0.168	**18.8 (9.0, 29.6)**	< 0.001*	**0.86**	**0.90**
**Left quadriceps**	1.2 (− 7.4, 9.7)	0.784	**11.4 (2.9, 20.6)**	**0.028**	**0.39**	**0.91**
**Right hamstrings**	− 2.2 (− 10.2, 5.6)	0.564	**17.4 (7.7, 28.2)**	< 0.001*	**0.98**	**0.93**
**Left hamstrings**	1.9 (− 7.1, 11.0)	0.666	**14.4 (3.3, 26.3)**	0.008*	**0.65**	**0.89**
**Right tibialis anterior**	**10.7 (0.8, 21.2)**	**0.030**	**28.5 (15.9, 42.7)**	< 0.001*	**0.82**	**0.87**
**Left tibialis anterior**	4.1 (− 7.9, 16.5)	0.180	**20.9 (6.0, 37.2)**	0.004*	**0.33**	**0.86**
**Right gastrocnemius/soleus**	5.6 (− 3.2, 14.7)	0.200	**18.9 (8.0, 30.6)**	< 0.001*	**0.64**	**0.83**
**Left gastrocnemius/soleus**	0.5 (− 8.2, 9.1)	0.911	**18.7 (8.0, 30.3)**	< 0.001*	**0.78**	**0.88**
Muscle-of-onset	− 0.08 (− 16.1, 16.0)	0.698	16.6 (− 2.9, 37.3)	0.070	0.77	0.73
**Region-of-onset**	2.7 (− 4.3, 9.7)	0.437	**14.1 (5.5, 23.2)**	0.001*	**0.87**	**0.89**
**Summary mean**	− 0.1 (− 6.2, 5.9)	0.972	**8.8 (1.3, 16.4)**	**0.017**	**0.36**	**0.56**

Significant T2-weighted radiological changes in MND patients between baseline and subsequent 4 and 12 month time-points are highlighted in bold. Results surviving multiple comparisons correction are asterisked

*CI* confidence interval*, SRM* standardized response mean, *Var* variance

