# Supplementary material for: Longitudinal multi-modal muscle-based biomarker assessment in motor neuron disease
Source: J Neurol. 2019 Oct 17;267(1):244–56. doi: 10.1007/s00415-019-09580-x (PMC6954906; doi:10.1007/s00415-019-09580-x)
Supplement: Supplementary file 1 — Supplementary file1 (DOCX 107 kb) [file 415_2019_9580_MOESM1_ESM.docx]

**SUPPLEMENTAL MATERIAL**

TABLE S1 Anatomical contouring landmarks

| **Structure** | **Acquisition station** | **Bone reference** | **Anatomical landmarks** |
| --- | --- | --- | --- |
| Tongue | Head/neck | C2 vertebra | The axial slice with widest diameter of tongue visible just above mandibular dentition was selected. |
| Sternocleido-  mastoids | Head/neck | C2 vertebra | The most prominent lateral muscle bulk, usually 3-4 axial slices below the level of the tongue was selected; right and left muscles contoured at same level. |
| Splenius capitis | Head/neck | C2 vertebra | The U-shaped muscle bulk just deep to trapezius, at the same level as sternocleidomastoids on the axial slice, was contoured. |
| Trapezius | Head/neck* | C2 vertebra* | The axial slice with widest diameter of muscle bulk visible just superior to the clavicle was selected. |
| Deltoids | Thorax* | Humerus* | Immediately inferior to the head of the humerus and superior to the level of the fascia separating deltoid from tricipital and teres minor, the anterolateral muscle bulk on axial slice was sampled. |
| Biceps brachii | Thorax | Humerus | Moving through axial slices superior to inferior, the muscle bulk immediately anterior to the humerus was sampled when visible at maximal diameter at the point where the humerus and the fascia dividing biceps and triceps were visible bilaterally. |
| Triceps | Thorax | Humerus | Moving through coronal slices anterior to posterior the maximal diameter muscle bulk of triceps was selected lateral to the humerus. |
| Forearm compartment encompassing brachioradialis | Abdomen | Distal ulna | Moving through axial slices inferior to superior, the maximal diameter muscle bulk with best defined boundaries medial to radius and ulna was contoured. |
| First dorsal interosseous | Abdomen | Distal ulna | Moving through coronal slices anterior to posterior, the maximal muscle bulk between first and second metacarpals was contoured. |
| Thenar eminence | Abdomen | Distal ulna | Moving through coronal slices anterior to posterior, the maximal diameter of the thenar eminence was identified, typically immediately posterior to first dorsal interosseous and adjacent to metacarpal bone. |
| Hypothenar eminence | Abdomen | Distal ulna | Moving through coronal slices anterior to posterior the maximal muscle, of the hypothenar eminence was identified and contoured adjacent to the fifth metacarpal. |
| Thoracic paraspinals | Thorax | Humerus | Moving through axial slices superior to inferior, at the level of the heart, the maximal muscle bulk was contoured, typically on the slice immediately superior to the upper border of the liver. |
| Psoas major | Abdomen | Distal ulna | Moving through axial slices superior to inferior, the maximal muscle bulk was contoured at the point where the transverse processes of the lumbar vertebrae were widest in anterior-posterior direction. |
| Gluteus maximus | Abdomen | Distal ulna | Moving through axial slices superior to inferior, the inferior limit of the bladder was identified and the muscle contoured at this level. |
| Quadriceps group | Upper leg | Femur | The axial slice was selected at the maximal diameter of both quadriceps and hamstrings. Contouring was performed around the femur, avoiding fascia separating vasti from sartorius. |
| Hamstrings group | Upper leg | Femur | The muscles were contoured at the same axial level as quadriceps, avoiding fascia and adductor groups. |
| Tibialis anterior | Lower leg | Tibia | The maximal anterolateral muscle bulk was contoured, excluding the peroneal muscle groups, at the calf level where gastrocnemius was of maximal diameter. |
| Gastrocnemius and soleus | Lower leg | Tibia | The posterior muscle group was contoured at the same level as tibialis anterior, selecting the maximal bulk. |
| C2 vertebra | Head/neck |  | The C2 vertebra was contoured at the level of the dens. |
| Humerus | Thorax |  | The slice containing the maximal diameter of the humeral head in continuity with the shaft was selected, of corresponding laterality to the muscles. |
| Distal ulna | Abdomen |  | The distal ulna was contoured at the slice of maximal diameter, of corresponding laterality to the muscles. |
| Femur | Upper leg |  | The slice containing the maximal diameter and length of femur was selected. |
| Tibia | Lower leg |  | At the level of the condyles and tuberosity, the slice containing the maximal diameter and length of tibia was selected. |

*In 2 participants, due to height, trapezius was contoured in the thoracic station and, in 3 participants, deltoid was contoured in the head/neck station.
